# Short-Term, Intermittent Fasting Induces Long-Lasting Gut Health and TOR-Independent Lifespan Extension

**DOI:** 10.1016/j.cub.2018.04.015

**Published:** 2018-06-04

**Authors:** James H. Catterson, Mobina Khericha, Miranda C. Dyson, Alec J. Vincent, Rebecca Callard, Steven M. Haveron, Arjunan Rajasingam, Mumtaz Ahmad, Linda Partridge

**Affiliations:** 1 Institute of Healthy Ageing, Genetics, Evolution and Environment, University College London, Darwin Building, Gower Street, London WC1E 6BT, UK; 2 Max Planck Institute for Biology of Ageing, Joseph-Stelzmann-Strasse 9b, 50931 Cologne, Germany

**Keywords:** intermittent fasting, dietary restriction, lifespan extension, *Drosophila melanogaster*, gut health, TOR independent, long lasting, memory effect, “2:5” diet, gut microbiota

## Abstract

Intermittent fasting (IF) can improve function and health during aging in laboratory model organisms, but the mechanisms at work await elucidation. We subjected fruit flies (*Drosophila melanogaster*) to varying degrees of IF and found that just one month of a 2-day fed:5-day fasted IF regime at the beginning of adulthood was sufficient to extend lifespan. This long-lasting, beneficial effect of early IF was not due to reduced fecundity. Starvation resistance and resistance to oxidative and xenobiotic stress were increased after IF. Early-life IF also led to higher lipid content in 60-day-old flies, a potential explanation for increased longevity. Guts of flies 40 days post-IF showed a significant reduction in age-related pathologies and improved gut barrier function. Improved gut health was also associated with reduced relative bacterial abundance. Early IF thus induced profound long-term changes. Pharmacological and genetic epistasis analysis showed that IF acted independently of the TOR pathway because rapamycin and IF acted additively to extend lifespan, and global expression of a constitutively active S6K did not attenuate the IF-induced lifespan extension. We conclude that short-term IF during early life can induce long-lasting beneficial effects, with robust increase in lifespan in a TOR-independent manner, probably at least in part by preserving gut health.

## Highlights

• The IF “2:5 diet” during early adulthood robustly extends lifespan in *Drosophila*
• Pro-longevity effects of IF are not dependent on reduced fecundity or food intake
• Early-life IF ameliorates late-life gut pathology and improves gut barrier function
• Epistasis experiments indicate a TOR-independent mechanism of action of IF

## Graphical Abstract

**Figure:**
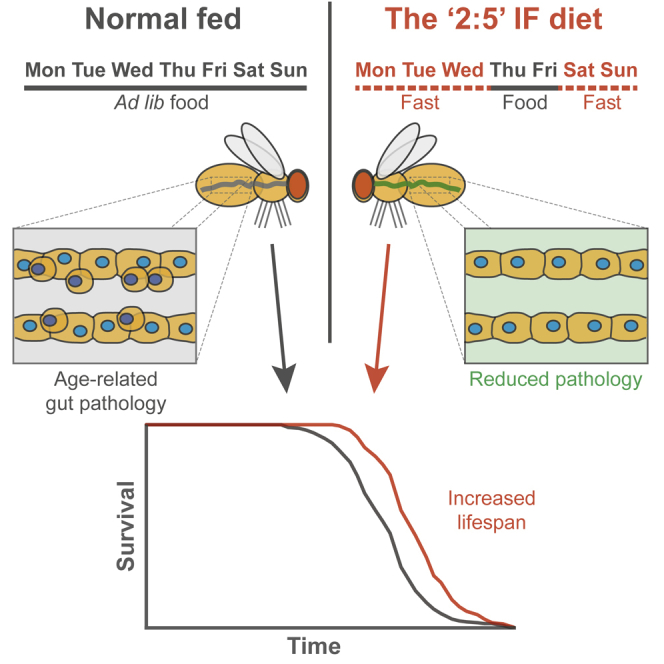

## Teaser

Intermittent fasting (IF) improves health and extends longevity in diverse model organisms. The fruit fly appeared to be the exception. Catterson et al. find that IF in early adulthood increases healthy lifespan of fruit flies. The effects of short-term IF are long-lasting, indicating that even brief IF periods may have lifelong health benefits.

## Introduction

Intermittent fasting (IF), an umbrella term for diets that cycle between a period of fasting and non-fasting, has become increasingly popular as a weight loss regime (e.g., “every-other-day fasting” and the “5:2” diet) [1, 2]. Advocates of IF argue that it shows many of the benefits seen with traditional daily energy restriction diets but with a simplified nutritional regime and increased compliance [3]. One study on the clinical outcomes of fasting in young overweight women described significant weight loss as a result of the IF regime, as well as reduced fat mass and waist circumference, and lowered serum cholesterol, triglycerides, and C-reactive protein [4]. More recent, pilot, clinical trials used a fasting mimicking diet (FMD) (consisting of monthly cycles of a 5-day fast during which daily food intake was reduced to ∼50% normal caloric intake), which reduced multiple health risk factors during the post-fast recovery period, including lowered blood pressure, and reduced blood glucose and insulin-like growth factor-1 (IGF-1) levels [5, 6]. However, systematic reviews of the clinical benefits of fasting regimens in humans found that study designs were heterogeneous and compliance data limited, making it difficult to draw definitive conclusions [7, 8].

IF can extend lifespan in a variety of organisms, including bacteria, yeast, nematode worms, and mice [9]. In animal models, IF has been shown to reduce the risk of developing a variety of age-related pathologies [9, 10]. IF is effective in preventing neurodegeneration in rodents [11, 12, 13, 14, 15] and can attenuate cancer [16] and cardiometabolic diseases, such as type II diabetes [4, 17, 18, 19, 20, 21]. FMD was recently found to increase pancreatic β cell regeneration in mouse models of diabetes [22].

DR, a chronic reduction of food intake without malnutrition, is an evolutionarily conserved method of improving health during aging and extending lifespan [23]. However, many studies of DR, particularly in rodents, also involve intermittent access to food, with the DR animals gorging their reduced meal as soon as it is supplied, leaving extended periods of time in a fasted state [24]. The beneficial health effects seen in DR may therefore be attributable, at least in part, to intermittent starvation. Supporting this idea, performing DR without the extended fasting periods by diluting the food of the DR mice with non-digestible cellulose, thus restricting total energy intake but allowing constant access to food, did not extend lifespan compared to fully fed animals [25]. In contrast, many studies in invertebrate organisms, including yeast, worms, and *Drosophila*, where the DR treatment involves continuous access to diluted food, result in robust lifespan extension [26]. Therefore, periodic fasting may be important for, but is likely not the only contributor to, the pro-longevity effects of DR, at least in these invertebrates.

Reduced activity of nutrient-sensing pathways, with corresponding decrease in global protein translation, is implicated as an important mechanism underlying the pro-longevity effects of dietary interventions, such as DR [23]. Reduced TOR signaling is a hallmark of pro-longevity interventions, including DR, and treatment with the TOR inhibitor rapamycin extends healthy lifespan in a range of organisms [27]. Although DR may exert some of its pro-longevity effects through reduced fecundity, DR can still extend lifespan in sterile, *ovo*^*D*^ mutant *Drosophila*, implying that fecundity and lifespan can be uncoupled and that other mechanisms are also important [28]. A recent study highlighted the importance of gut homeostasis in DR-induced longevity in *Drosophila*, because DR both rescued age-related gut pathologies and extended lifespan in females and in males with feminized guts, but not in wild-type males, which do not undergo significant age-related gut pathology [29]. Diet composition and fly food transfer schedule are also known to affect the abundance and diversity of fly-associated bacteria [30], and periods of fasting may be expected to modulate the associated microbiota.

Invertebrate model organisms provide powerful contexts in which to establish the molecular mechanisms mediating the pro-longevity effects of IF. In yeast, Rim15, a key integrator of signals transduced by the Sch9, Ras, and TOR pathways, is important for starvation-induced lifespan extension [31] while in the nematode worm Rheb-1, with a key role in TOR signaling, is essential for the pro-longevity effects of IF [32]. Interestingly, the findings with both organisms imply the existence of additional underlying mechanisms mediating the pro-longevity effects of IF. It is unclear whether Rim15/Rheb-1 and their interactors mediate the effects of IF in mammals.

Previous studies examining potential pro-longevity effects of IF in flies have produced mainly negative results [26, 33, 34]. The first studies, almost 90 years ago, found that 6 hr of starvation in every 24 hr was beneficial and could extend lifespan [34]. However, the effects of this IF regime may be strain or food medium specific, because a similar, more rigorous experiment ∼80 years later found that daily bouts of either 3 hr or 6 hr starvation throughout the adult life of the fly had neither a positive nor a negative effect on lifespan [26]. Interestingly, short-term fasting can increase resistance to severe cold stress [35] and facilitate long-term memory formation in flies [36]. Time-restricted feeding (TRF) in *Drosophila* improves sleep consolidation as well as a variety of cardiac output functions that normally decline with age, despite the caloric ingestion/expenditure of TRF flies being the same as *ad-libitum*-fed flies [37]. Thus, health improvements can result from various IF regimes in *Drosophila*, but the mechanisms at work await elucidation and the evidence for a pro-longevity phenotype from IF is more mixed.

Here, we investigated a variety of IF regimes in flies and their effects on a range of health outcomes, including feeding behavior, gut and metabolic health, survival after stress, and lifespan. Importantly, short-term IF (the “2:5” diet) confined to early life robustly increased subsequent lifespan, particularly in females, independent of TOR signaling. Short-term IF also led to long-lasting health improvements, including increased stress resistance and a lower incidence of gut pathology that was associated with reduced bacterial abundance.

## Results

### IF during Early Life Extends Lifespan

**Figure 1:**
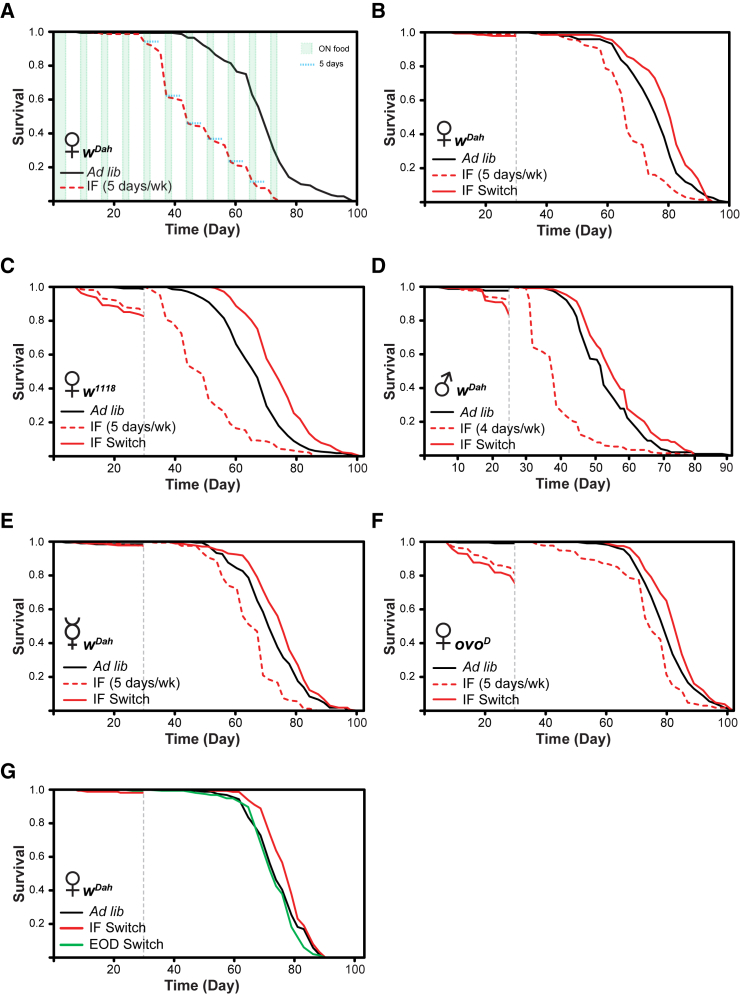
IF during Early Life Extends Lifespan and Is Not Dependent on Reduced Fecundity (A) Fasting for 5 consecutive days per week significantly (p = 2.4 × 10^−28^; log rank test) shortened lifespan compared to *ad libitum* controls. n > 150 flies per condition. (B) Switching 5-day-fasted flies to *ad libitum* after 30 days of the IF regimen resulted in significantly increased lifespan compared to *ad libitum* controls (p = 0.0022; log rank test; n > 145 flies per condition). (C) Isogenic *w*^*1118*^ flies exhibited a greater sensitivity to 5-day IF compared to outbred *w*^*Dah*^ flies (see A and B). Switching 5-day IF *w*^*1118*^ flies to *ad libitum* after 30 days of the regime resulted in significant lifespan extension compared to *ad libitum* controls (p = 3.0 × 10^−10^; log rank test). n > 180 flies per condition. (D) Switching male flies fasted for 4 consecutive days per week after 25 days of the regime significantly increased lifespan compared to *ad libitum* controls (p = 0.0094; log rank test). n > 150 flies per condition. (E) Switching 5-day IF virgin female flies to *ad libitum* after 30 days of the regime resulted in significant lifespan extension compared to *ad libitum* controls (p = 0.012; log rank test). n > 170 flies per condition. (F) Sterile *ovo*^*D*^ flies exhibited a greater sensitivity to 5-day IF compared to outbred *w*^*Dah*^ flies (see A and B). Switching 5-day IF *ovo*^*D*^ flies to *ad libitum* after 30 days of the regime resulted in significant lifespan extension compared to *ad libitum* controls (p = 0.0048; log rank test). n > 160 flies per condition. (G) Every-other-day fasting for the first 30 days of life (EOD switch) did not affect lifespan compared to *ad libitum* controls (p = 0.096; log rank test), whereas IF switch significantly increased lifespan compared to *ad libitum* controls (p = 0.017; log rank test). n > 166 flies per condition. Grey dashed line indicates the “switch” point at 30 days (25 days for males), after which the lifespan curves were “reset” and deaths before this point were censored. See also Figures S1, S2, S3, and S4.

We hypothesized that the periods of starvation in previously published IF regimes may have been insufficient in duration to influence survival [26]. We therefore began by fasting female flies by exposing them to nutrient-free agar gel for 5 consecutive days per week (5-day IF—the 2:5 diet) throughout life and found that this led to earlier onset of age-related mortality and significantly shortened lifespan compared to *ad-libitum*-fed controls (Figure 1A). Mortality rates were lower during 2-day feeding periods in the IF flies, an effect that persisted into the first 3 days after food was withdrawn. Because 5 days of continuous starvation was detrimental, we next investigated the effects of 5 days non-continuous starvation per 7 days by fasting for 3 days, feeding for 1 day, fasting for 2 days, and feeding for 1 day and found that this regime also significantly shortened lifespan, although with a mildly protective effect up to day 50 (Figure S1A). Taken together with the observation that 5-day continuous IF did not start to induce mortality until the flies were ∼30 days old (Figure 1A), these findings suggested that the effects of IF might be age specific. Fly feeding rate declines with age [38], and older flies may therefore be more sensitive to starvation and exhaustion of energy stores. Any beneficial effect of IF may therefore be lost or even reversed later in life.

To test directly for an age-specific benefit of IF, we repeated the 5-day continuous IF experiment and included an additional IF group that was returned to *ad libitum* feeding after 30 days of the IF regime (labeled “IF switch”). Lifespan was again shortened by 5-day continuous IF throughout life (Figure 1B), but the IF switch group had significantly extended lifespan compared to the *ad libitum* controls. An independent repeat of this experiment confirmed these results (Figure S1B). Thus, continuous exposure to the 2:5 diet induced mortality in older flies, but the same dietary regime for the first 30 days of adulthood resulted in significant lifespan extension in female flies, indicating a long-lasting “memory” effect of this early-life intervention.

Less severe IF regimes might also result in positive outcomes compared to 5-day continuous IF. We therefore next examined the effects of shorter fasting duration periods with 2-day, 3-day, and 4-day continuous IF throughout adult life. None of these regimes had a significant effect on lifespan (Figures S2A–S2C). When the same regimes with an additional IF switch condition were examined, 2-day and 4-day IF switch had no significant effect, whereas the 3-day IF switch condition lived significantly longer (Figures S2D–S2F). Increasing the caloric content of the food is another way in which the potential severity of IF may be offset. When flies were fed food with twice the amount of yeast (“2SY”) [39], fasting for 2 days per week throughout adult life significantly increased lifespan (Figure S2G).

To rule out strain specificity of our results, we performed IF on the isogenic lab strain *w*^*1118*^. Lifespan was significantly extended in *w*^*1118*^ after 30 days of 5-day IF (Figure 1C). *w*^*1118*^ females initially exhibited a greater sensitivity to 5-day IF compared to outbred *w*^*Dah*^ flies (Figures 1A and 1B), and so, to rule out a selection bias for the most starvation-resistant flies, we also applied shorter duration IF to these flies. Whereas 3-day IF switch had no significant effect (Figure S3A), 4-day IF switch significantly extended lifespan in *w*^*1118*^ flies (Figure S3B). Importantly, the early-life mortality disappeared in these flies, whereas the longevity phenotype remained.

Male flies also benefited from the IF switch regime. Male flies are more starvation sensitive than females [40]. Therefore, males were exposed to 4-day IF for 25 days. This resulted in significant lifespan extension (Figure 1D). We also tested 2-day IF and 3-day IF on males, but there was no effect (Figures S3C and S3D). Therefore, both sexes can benefit from early-life dietary intervention with increased longevity, and these effects are dependent on the duration of the fasting period.

### Extension of Lifespan by IF Is Not Mediated by Reduced Fecundity

IF induced a greater lifespan extension in female flies compared to males. We hypothesized that this may have been due to reduced fecundity, a hallmark of pro-longevity interventions, such as DR [26]. We therefore performed egg counts during the fasting/fed period. Fecundity was indeed all but abolished during the fasting period and was restored after re-feeding (Figure S1C).

Because the 2:5 diet reduced egg production, we experimentally tested the role of reduced fecundity in the increased longevity from IF. We performed IF on virgin females, which produce fewer eggs than mated females [41], and on sterile females carrying the dominant *ovo*^*D*^ mutation, which blocks oogenesis [42]. Lifespan was significantly extended in virgin *w*^*Dah*^ females after IF (Figure 1E). Additionally, whereas *ovo*^*D*^ females initially exhibited a greater sensitivity to 5-day IF compared to outbred *w*^*Dah*^ flies (see Figures 1A and 1B), 5-day IF switch *ovo*^*D*^ flies lived significantly longer than controls (Figure 1F). To rule out a selection bias for the most starvation-resistant *ovo*^*D*^ flies, we also performed shorter duration IF on these flies. Whereas 3-day IF switch in *ovo*^*D*^ flies had no significant effect on lifespan (Figure S3E), 4-day IF switch significantly extended lifespan (Figure S3F) but without any excess early mortality. Therefore, similar to DR [43], the pro-longevity phenotype observed after short-term early-life IF is not mediated by reduced fecundity.

### Effects of IF Are Duration and Age Specific

**Figure 2:**
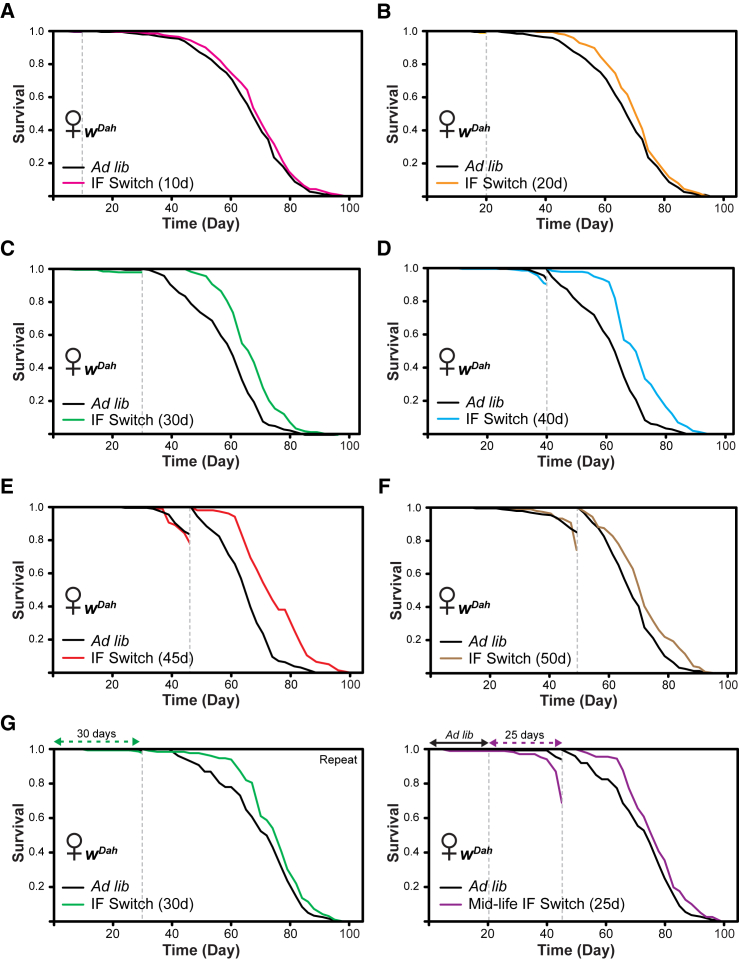
Modulation of 5-Day IF Regime Duration in Females (A) Switching 5-day IF flies to *ad libitum* after 10 days of the regime did not affect lifespan compared to *ad libitum* controls (p = 0.15; log rank test). n > 195 flies per condition. (B) Switching 5-day IF flies to *ad libitum* after 20 days of the regime did not affect lifespan compared to *ad libitum* controls (p = 0.057; log rank test). n > 195 flies per condition. (C) Switching 5-day IF flies to *ad libitum* after 30 days of the regime resulted in significant lifespan extension compared to *ad libitum* controls (p = 3.6 × 10^−10^; log rank test). n > 190 flies per condition. (D) Switching 5-day IF flies to *ad libitum* after 40 days of the regime resulted in significant lifespan extension compared to *ad libitum* controls (p = 1.2 × 10^−13^; log rank test). n > 165 flies per condition. (E) Switching 5-day IF flies to *ad libitum* after 45 days of the regime resulted in significant lifespan extension compared to *ad libitum* controls (p = 1.4 × 10^−14^; log rank test). n > 150 flies per condition. (F) Switching 5-day IF flies to *ad libitum* after 50 days of the regime resulted in significant lifespan extension compared to *ad libitum* controls (p = 0.00046; log rank test). n > 145 flies per condition. (G) Switching 5-day IF flies to *ad libitum* after 30 days of the regime resulted in significant lifespan extension compared to *ad libitum* controls (p = 0.019; log rank test). Switching flies from *ad libitum* food for the first 20 days of life to 5-day IF spanning days 20–45 (25 days) and then back to *ad libitum* food resulted in significant lifespan extension compared to *ad libitum* controls (p = 0.045; log rank test). n > 145 flies per condition. Grey dashed line indicates the switch point, after which the lifespan curves were reset and deaths before this point were censored. For display purposes, in (A), (B), and (F), the *ad libitum* condition is the same data in each separate graph (solid black line). In (C), (D), and (E), the *ad libitum* condition is the same data in each separate graph. In (G), the *ad libitum* condition is the same data in each separate graph. See also Figure S3.

Every-other-day (EOD) fasting involves alternating feeding/fasting on consecutive days and is less severe than 5 days of continuous fasting. We performed EOD fasting for the first 30 days of life (EOD switch) and found no effect on lifespan compared to *ad libitum* controls, whereas the 5-day IF switch significantly increased lifespan (Figure 1G). We next investigated the optimum duration of early-life IF for extension of lifespan in female flies. Neither 10 nor 20 days of 5-day IF extended lifespan (Figures 2A and 2B), whereas 30 days (Figure 2C), 40 days (Figure 2D), and 45 days of 5-day IF (Figure 2E) all did. 50 days of 5-day IF slightly reduced lifespan extension compared to 45 days (Figure 2F). Additionally, 50 days of the IF diet led to some early death before the IF switch to *ad libitum* conditions. Therefore, there appears to be a duration-specific and age-specific window where IF can exert its beneficial effects.

### IF Restricted to Mid-life IF Can Also Extend Lifespan

To assess the effects of IF at stages other than during early adulthood, we also examined the effect of a “mid-life” and “late-life” IF on lifespan. We again observed significant lifespan extension in 5-day IF switch flies after 30 days of the regime in early adulthood (Figure 2G). To perform a mid-life IF switch, flies were kept on *ad libitum* food for the first 20 days of adulthood, moved to 5-day IF from days 20 to 45 (25 days of the regime), and then placed back on *ad libitum* food for the remainder of adult life. We observed a significant lifespan extension in mid-life IF switch flies compared to *ad libitum* controls, although there was notable mortality before the switch back to *ad libitum* food. We next assessed the effect of late-life IF on lifespan. Switching *w*^*Dah*^ flies to 5-day IF after 30 days of *ad libitum* food significantly shortened lifespan (Figure S3G), indicating the pro-longevity effects of IF are exerted during early life and, to a lesser extent, mid-life.

### IF Increases Stress Resistance

Most pro-longevity interventions in flies are accompanied by increases in stress responses [44], including starvation resistance. We performed a starvation assay on 42-day-old post-IF females (Figure S3H) and found that flies previously exposed to IF lived significantly longer. We next examined the effects of dichlorodiphenyltrichloroethane (DDT), a xenobiotic toxin, and paraquat, an oxidative stress, on the survival of 42-day-old post-IF females (Figures S3I and S3J). From two independent replicate studies, post-IF flies exhibited a small but significantly enhanced resistance to DDT and paraquat. Therefore, early IF can lead to long-lasting stress resistance. Post-IF flies that were fed DDT and paraquat exhibited increased resistance to these toxins, and we hypothesized that the protective effects of IF may be due, at least in part, to improvements in gut health. In contrast, although DR is associated with increased TAG and starvation resistance [45], DR does not increase xenobiotic resistance in flies, and thus IF is distinct from DR in this respect [46].

### Cumulative Food Intake Is Not Reduced during IF and Increases after the IF Switch

Flies exposed to the 5-day IF regime are fed for 2 days per week. We hypothesized the subsequent lifespan extension observed in IF switch flies could be due to an overall lifelong reduction in calorie intake, as in DR. We therefore examined the effect IF had on total food intake by performing the quantitative capillary feeder (CAFE) assay (Figures S4A–S4C). During the 2-day feeding period, 5-day IF flies ingested significantly more food than controls (Figure S4A). However, after 14 days, there was no significant difference in cumulative food ingestion between IF flies and controls (Figures S4B and S4C).

We also examined post-IF feeding behavior using the CAFE assay and began measuring at 6 days after the IF switch. Cumulative food intake was significantly higher in IF flies compared to *ad libitum* controls (Figure S4D). We conclude that cumulative food intake was not significantly reduced during IF, whereas feeding was increased after IF. Therefore the IF-induced lifespan extension was not due to a DR-like reduction in total feeding post-IF.

### IF Lowers Lipid Content, with Recovery Post-IF

Fasting triggers mobilization of internal fat stores, which can enable animals to survive during extended periods of starvation. We therefore performed a time course experiment measuring whole-fly triacylglyceride (TAG) from 5-day IF switch flies. We also examined flies that remained on the 5-day IF regime. Under *ad libitum* conditions, TAG levels increased with age and plateaued around day 37 (Figure S4E). In contrast, 5-day IF flies had significantly reduced TAG levels when fully fasted. On day 37, TAG levels in IF switch flies had not fully recovered to *ad libitum* levels. However, TAG levels in IF switch flies recovered to *ad libitum* levels from day 46 (i.e., 2 weeks after the switch to *ad libitum* food). We examined TAG levels in 60-day-old females (i.e., 30 days since the end of IF diet). Whole-fly TAG levels were significantly higher in IF switch flies compared to *ad libitum* controls (Figure S4G). TAG levels at day 60 from 3-day, 4-day, and 5-day IF switch flies were also examined. TAG levels were significantly higher in 5-day IF switch flies alone compared to the other conditions (Figure S4H), highlighting a likely explanation for the greater pro-longevity effect of 5-day IF switch compared to 3- or 4-day IF switch.

Whole-fly protein levels were not significantly different between any of the conditions tested (Figures S4F–S4H). Therefore, the reduction in TAG levels during the 5-day IF period recovered and stabilized to *ad libitum* levels post-IF. Indeed, it appears that TAG was accumulating in 5-day IF switch flies. Increased TAG levels at 60 days may indicate a mechanism behind the long life of IF switch flies.

### IF Preserves Gut Homeostasis and Is Associated with Reduced Bacterial Abundance

Maintenance of gut homeostasis has been shown to play an important function in the determination of lifespan in *Drosophila* [29, 47, 48]. Midgut tissue homeostasis is maintained by intestinal stem cells (ISCs), which are arranged along the basement membrane and respond to stress and lead to a regenerative response. In the aging female fly gut, ISC proliferation becomes dysregulated and can lead to mis-differentiation and hyperplasia in the intestine. DR reduces gut pathology in aging females [29], and we therefore sought to examine the effects of IF on gut homeostasis.

**Figure 3:**
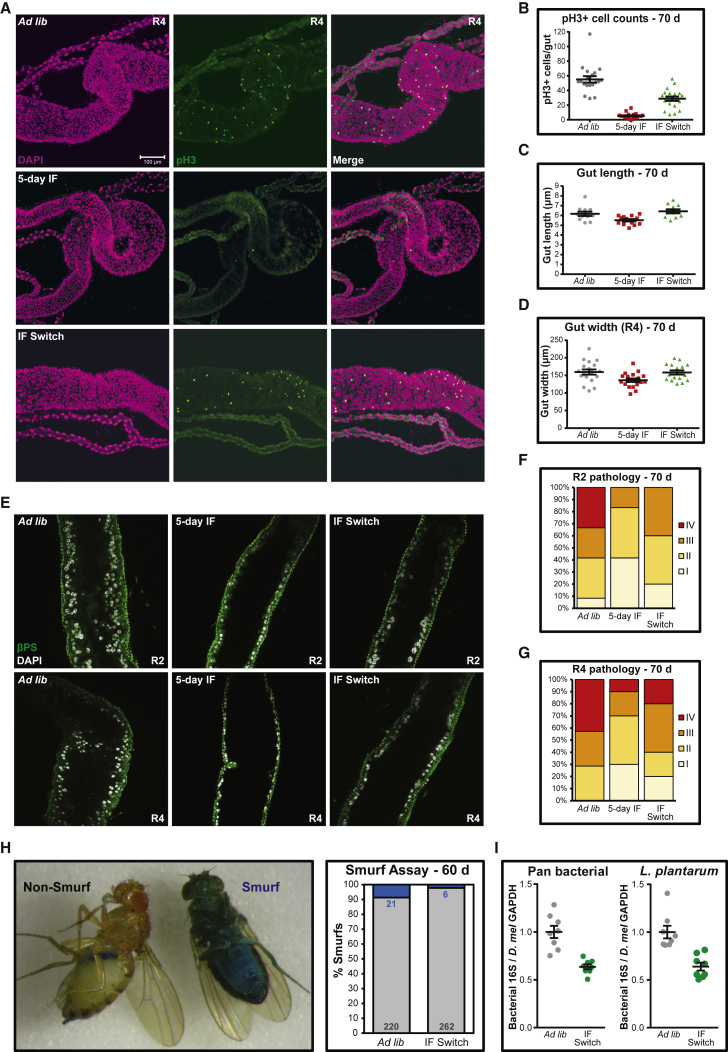
Early-Life Fasting Leads to Long-Lasting Improvements in Gut Health and Reduced Bacterial Load (A) ISC activity. Representative confocal fluorescence z projections of midgut regions 4/5 from 70-day-old *ad libitum*, 5-day IF, and IF switch flies stained with DAPI (magenta) and anti-phospho-H3 antibody (pH3, green) are shown. The scale bar represents 100 μm. (B) Quantification of pH3-positive (pH3+) cells per gut. After 5 days of fasting, 70-day-old 5-day IF guts had significantly fewer pH3+ cells compared to *ad libitum* controls (p < 0.0001; one-way ANOVA). IF switch guts, after 40 days on food, also had significantly fewer pH3+ cells compared to *ad libitum* controls (p < 0.0001; one-way ANOVA). 19 or 20 guts per condition are shown. Data are shown as mean ± SEM. (C) Quantification of gut length. After 5 days of fasting, 70-day-old 5-day IF guts were significantly shorter than *ad libitum* controls (p = 0.038; one-way ANOVA). IF switch guts were not significantly different from *ad libitum* controls. 11–13 guts per condition are shown. Data are shown as mean ± SEM. (D) Quantification of gut width at R4 section. After 5 days of fasting, 70-day-old 5-day IF guts were significantly narrower than *ad libitum* controls (p = 0.025; one-way ANOVA). IF switch guts were not significantly different compared to *ad libitum* controls. 16–18 guts per condition are shown. Data are shown as mean ± SEM. (E) Midgut pathology. Representative confocal fluorescence sections of R2 (top row) and R4 (bottom row) midgut regions from 70-day-old *ad libitum*, 5-day IF, and IF switch flies stained with DAPI (white) to visualize nuclei and anti-βPS-integrin antibody (βPS, green) to visualize the basement membrane/muscle are shown. (F and G) Pathologies were binned into scaled categories and quantified. R2 (F, n = 10–12 per condition) and R4 (G, n = 7–10 per condition) categories were defined as follows: I, wild-type (WT), nuclei form single layer epithelium; II, sporadic pathology of small nuclei “nests”; III, intermediate pathology, sections of epithelium have several layers of nuclei; and IV, widespread pathology, majority of epithelium has several layers of nuclei. (H) Barrier function at day 60, as measured by “smurf” flies with leaky intestines, was significantly improved in IF switch females compared to *ad libitum* controls (p = 0.0013; Fisher’s exact; n numbers on panel). (I) Quantification of relative bacterial abundance in whole females by qPCR of the bacterial 16S rRNA gene with pan-bacterial primers and *Lactobacillus*-*plantarum*-specific primers. IF switch flies at day 40 (i.e., day 10 post-IF) had significantly reduced bacterial abundance compared to *ad libitum* controls (pan bacterial, p = 0.00012; *L. plantarum*, p = 0.0004; Student’s t test). Values were normalized to the *Drosophila* GAPDH gene. Data are shown as mean ± SEM (n = 8 biological replicates per condition).

We examined the R4 midgut region from 70-day-old females and compared *ad libitum*, 5-day IF, and IF switch conditions. Mitotically active ISCs were visualized by phospho-histone H3 (pH3) immunostaining. *Ad-libitum*-fed females exhibited widespread ISC activity, whereas fully fasted 5-day IF females had significantly fewer pH3-positive cells (Figures 3A and 3B), indicating ISC quiescence. Notably, after 40 days since the switch to *ad libitum* food, IF switch flies still had significantly fewer pH3-positive cells compared to *ad libitum* controls. Gut length was significantly shorter in fully fasted 5-day IF females compared to *ad libitum* controls (Figure 3C). IF switch guts, however, were not shorter than those of *ad libitum* controls. We also measured the widths of R4 regions from each gut (Figure 3D) and found that, after 5 days of fasting, 70-day-old 5-day IF guts were significantly narrower than *ad libitum* controls. IF switch guts were not narrower than *ad libitum* controls. Therefore, the age-related pathology of increased stem cell activity in old female guts, as measured by pH3 staining, was rescued by short-term early-life IF intervention. This protection appeared to be long lasting and was not due to major morphological or structural changes in the gut.

We next examined gut pathology in the R2 and R4 regions of the midgut from 70-day-old females and compared *ad libitum*, 5-day IF, and IF switch conditions. In R2/R4 regions of young female guts, nuclei are organized typically in a single-layer epithelium, whereas in aged females, the epithelium can exhibit several layers of nuclei, indicating widespread disruption and pathology [29]. Pathologies were scored blind, binned into scaled categories, and quantified. 70-day-old *ad libitum* females exhibited several layers of nuclei in both R2 and R4 midgut epithelia, indicating widespread pathology, whereas fully fasted 5-day IF females had reduced pathology, particularly in the R2 midgut region (Figures 3E–3G). Similar to ISC activity, IF switch flies had noticeably reduced R2 and R4 midgut pathology compared to *ad libitum* controls. This confirms the protective effect of short-term early-life IF on age-related tumor-like gut pathologies in females.

As a functional readout of gut homeostasis, we measured gut barrier function using the smurf assay [48]. In young flies, FD&C blue dye no. 1 does not normally pass through the gut into the body cavity, but in a small population of old flies, the blue dye can pass through the gut barrier and cause the flies to turn blue (or “smurf”). We found that there were significantly fewer 60-day-old IF switch flies that smurfed compared to *ad libitum* controls (Figure 3H), indicating improved gut barrier function due to IF.

Gut barrier integrity is linked to microbial dysbiosis with age [49, 50], and removal of bacteria in late adulthood extends lifespan [51]. Diet and fly food transfer schedule are also known to affect the diversity and abundance of fly-associated bacteria, and the fasting regime we perform may be expected to modulate the associated microbiota. We therefore compared relative bacterial abundance in 40-day-old (i.e., day 10 post-IF) whole females by qPCR of the bacterial 16S rRNA gene with pan bacterial and *Lactobacillus*-*plantarum*-specific primers. *L. plantarum* was chosen as it is one of the most abundant commensals in the adult fly, and it was also recently associated with loss of gut epithelial integrity in adults [52]. Relative bacterial abundance was significantly reduced in IF switch flies compared to *ad libitum* controls using both pan bacterial and *L. plantarum* primers (Figure 3I).

The 2:5 diet thus protected against the age-related deterioration of female gut homeostasis, as measured by reduced ISC activity without major ultrastructural changes, and, with reduced tumor-like pathologies in both R2/R4 regions, protected against the loss of gut barrier function and led to reduced bacterial load.

### IF Acts Independently of the TOR Pathway

We hypothesized that the beneficial effects of IF on lifespan were acting, at least in part, via the TOR pathway. IF appeared to phenocopy many of the beneficial effects on lifespan, stress resistance, and gut health observed with reduced TOR signaling [53, 54]. Indeed, reduced TOR signaling has been shown to prolong longevity in a variety of organisms [27]. Additionally, rapamycin can extend *Drosophila* lifespan beyond the maximum achieved by DR [53], indicating that the effects of rapamycin are partially independent of those of DR. Because the effects of rapamycin and DR on lifespan are additive, we examined whether IF and rapamycin were also additive.

**Figure 4:**
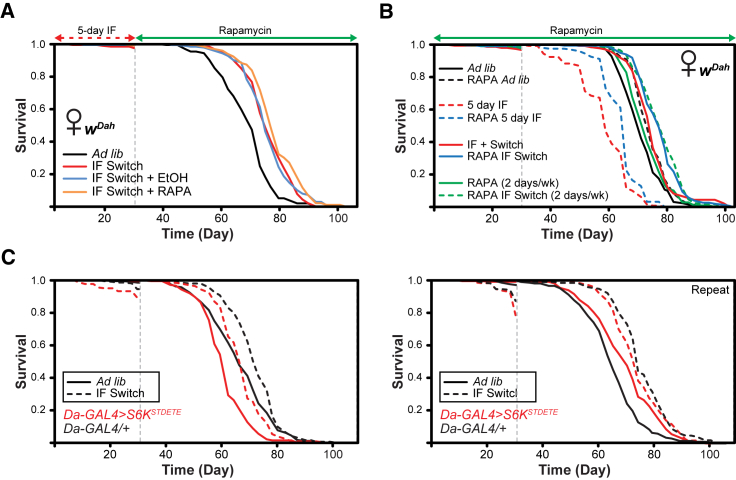
Genetic and Pharmacological Epistatic Analysis Reveals a TOR-Independent Mechanism for the Lifespan-Extending Effects of IF (A) IF switch and IF switch with EtOH (vehicle control, beginning after IF) extended lifespan compared to *ad libitum* controls (IF switch, p = 1.4 × 10^−13^, log rank test; IF switch + EtOH, p = 5.0 × 10^−13^, log rank test). Rapamycin treatment after IF (i.e., starting at 30 days) further extended lifespan compared to IF switch and IF switch + EtOH controls (Rapa versus IF switch, p = 0.0081; Rapa versus IF switch + EtOH, p = 0.013 log rank test). n > 200 flies per condition. (B) *Ad libitum* rapamycin treatment (black dashed line) extended lifespan compared to untreated controls (solid black line; p = 0.0003, log rank test). Rapamycin treatment during the “fed” period of the IF diet (blue dashed line) rescued the lifespan-shortening effects of the IF diet for the duration of the lifespan (red dashed line; p = 1.2 × 10^−07^; log rank test). IF switch (solid red line) extended lifespan compared to *ad libitum* controls (solid black line; p = 0.00018; log rank test). Rapamycin treatment during the fed period (i.e., 2 days per week) of the IF switch diet and then *ad libitum* sugar/yeast/agar (SYA) thereafter (solid blue line) led to a further significant increase in lifespan compared to the untreated IF switch condition (solid red line; p = 0.00078; log rank test). Rapamycin treatment for 2 days per week and *ad libitum* SYA the rest of the time (solid green line) led to a small but significant lifespan extension compared to untreated *ad libitum* controls (solid black line; p = 0.034; log rank test). Rapamycin treatment during the fed period (i.e., 2 days per week) of the IF switch diet and then *ad libitum* SYA thereafter with rapamycin treatment 2 days per week (green dashed line) led to a further significant increase in lifespan compared to the untreated IF switch condition (solid red line; p = 6.7 × 10^−05^; log rank test). Cox proportional hazards analysis of the lifespan demonstrated a significantly reduced risk of dying when flies were exposed to IF versus *ad libitum* (p = 6.95 × 10^−05^) or rapamycin versus *ad libitum* control food (p = 0.00055). There was no significant interaction between IF and rapamycin (p = 0.94), indicating IF and rapamycin had independent effects on lifespan. n > 143 flies per condition. (C) Two replicate lifespan experiments examining the effect of constitutive S6K activation on the pro-longevity effect of IF. IF led to significant lifespan extension in *Da-GAL4* driver-alone heterozygotes compared to *ad libitum* controls (left panel, p = 8.7 × 10^−05^; right panel, p = 7.7 × 10^−21^; log rank test). Ubiquitous overexpression of the constitutively active S6K (*UAS-S6K*^*STDETE*^) via *Da-GAL4* did not abolish the lifespan-extending effects of IF (left panel, p = 4.0 × 10^−12^; right panel, p = 0.0027; log rank test). There was no significant interaction between IF and S6K (p = 0.10), indicating IF and S6K had independent effects on lifespan. n > 200 flies per condition for both experiments.

We first examined the effect of administering rapamycin after 30 days of the IF regime and compared these with IF vehicle (EtOH) and IF controls (Figure 4A). We found that the lifespan-extending effects of rapamycin and IF were additive, indicating distinct mechanisms. Using a different experimental setup, instead of administering rapamycin after the early-life IF period, we administered rapamycin to flies throughout the entire lifespan (Figure 4B). Compared to untreated *ad libitum* controls, *ad libitum* rapamycin and IF switch both extended lifespan to a similar extent. During the IF regime, flies have access to food for 2 days per week, so to control for feeding fasted flies rapamycin during the 2-day “fed” period, we also added an *ad libitum* condition where we fed rapamycin only during the 2-day fed period. We observed a small but significant lifespan extension in flies fed rapamycin 2 days per week compared to untreated *ad libitum* controls. Rapamycin was protective against the deleterious effects of lifelong IF. Treatment with rapamycin during the 2 day fed period of the IF switch diet then *ad libitum* thereafter resulted in increased lifespan compared to the untreated IF switch condition. Similarly, for flies treated with rapamycin during the 2-day fed period of the IF switch diet then *ad libitum* rapamycin thereafter, lifespan was increased compared to the untreated IF switch condition. Compared to the already extended lifespans of IF switch or rapamycin treatments alone, flies that were exposed to both IF and rapamycin, whether with *ad libitum* food or *ad libitum* rapamycin during the post-IF period, further extended lifespan to a similar extent. Cox proportional hazards analysis of the lifespan found no significant interaction between IF and rapamycin, indicating IF and rapamycin have independent effects on lifespan. Therefore, the lifespan-enhancing effects of IF and rapamycin are additive, indicating non-overlapping mechanisms.

To further elucidate the mechanisms mediating the lifespan-extending effects of IF, we also performed genetic epistasis experiments. S6K facilitates the downstream effects of TOR by its effects on protein translation, and rapamycin-induced lifespan extension was previously shown to be abrogated by its upregulation [53]. We therefore examined the effects of IF on flies ubiquitously expressing a constitutively active form of S6K (*UAS-S6K*^*STDETE*^) [55]. In two independent experiments, we found that IF led to significant lifespan extension in *Da-GAL4* driver-alone heterozygotes compared to *ad libitum* controls, whereas ubiquitous overexpression of the constitutively active S6K did not abolish the lifespan-extending effects of IF (Figure 4C). Cox proportional hazards analysis of the pooled lifespan data found no significant interaction between IF and S6K. We can therefore conclude that IF is not epistatic with, and is acting independently of, TOR.

## Discussion

IF can be highly effective against age-related diseases, including diabetes, cancer, and cardiovascular disease [6]. Although IF research is still in its infancy, the mechanisms mediating the health improvements seen during and after IF are starting to be discovered.

In this study, we clarified the effects of IF on health and lifespan in *Drosophila*. In line with previous studies [26, 33], we found that IF for short fasting periods over the duration of the adult female lifespan had no beneficial effect (e.g., 2 fasted days per week). IF shortened lifespan when the fasting period was lengthened, e.g., 5 fasted days per week (the 2:5 diet). However, when flies were switched to *ad libitum* conditions after 30 days of a prolonged IF regime, median lifespan could be extended by as much as 10%. The pro-longevity effect of short-term, age-specific IF was not sex specific but was more effective in females, was consistent over a range of genotypes, was not due to a DR-like reduction in post-IF food intake, and was not dependent on reduced fecundity, indicating mechanisms other than reproductive trade-offs.

Remarkably, IF during early adulthood, and also during mid-life, was sufficient to extend lifespan, indicating a “memory” effect. Increased resistance to multiple stresses after IF was also an indication of significantly improved overall health. Hormesis is a phenomenon by which “low-level” toxic stress elicits response mechanisms that protect against similar but higher level stresses associated with aging [56]. Given that intermittent starvation in early life led to increased post-IF starvation resistance in addition to increased lifespan, hormesis could play a role. Although we cannot rule out the possibility of a hormetic effect of IF, the pro-longevity effect of IF restricted to early and mid-life, and the opposing lifespan-shortening effect of IF restricted to late life, suggests an age-specific effect of IF and that other mechanisms may be more relevant. Further work is necessary to elucidate the potential hormetic effects of IF, as well as assessment of longevity interventions during specific fly life stages, particularly early versus late interventions, as this remains a relatively underexplored area of aging research [57].

The increased TAG levels, stress resistance, and longevity are also potentially a direct consequence of post-IF hyperphagia. Intriguingly, 42-day-old IF switch flies were more starvation resistant compared to *ad libitum* controls but had lower TAG levels, indicating a mechanism other than increased fat storage. A more detailed study of feeding behavior during IF and post-IF would be necessary to investigate this hypothesis.

IF influenced the female gut, with a distinct improvement in measures of age-related pathology, including a preservation of the “youthful” phenotype, i.e., reduced ISC activity; fewer layers of nuclei, indicating reduced tumor formation; and improved gut barrier function [29]. However, gut length and gut width were no different from those of controls, suggesting a recovery of gut functionality without confounding effects of shorter/narrower guts, which may result in indirect (functional) DR. Indeed, the increased lipid content in 60-day-old flies despite early-life fasting points to a protective effect from the age-related decline in gut function. However, further analysis probing the absorptive capacity of the gut during aging is necessary before improved gut health and absorption can be linked. The increased TAG levels seen in 5-day IF switch flies, but not in 3- or 4-day IF switch flies, may also explain the differing effectiveness of each of these regimes.

Increased TAG is also observed with loss of fly-associated bacteria [58], which is consistent with the changes in TAG seen with IF switch flies. We therefore measured relative bacterial abundance in post-IF females and found that, despite higher post-IF food intake, relative bacterial abundance was reduced in IF switch flies. Longer periods of fasting appear to reduce total bacterial load, and this is associated with improved gut health and lifespan extension. However, further work is required to elucidate whether the beneficial effects of IF are due to reduced microbial load and should assess whether IF would lead to increased longevity in germ-free conditions.

Finally, we probed the mechanisms underlying the pro-longevity properties of IF. We performed pharmacological and genetic epistatic analysis to demonstrate that IF acts independently of the TOR pathway, because rapamycin and the IF regimen acted additively to extend lifespan. Similarly, Bjedov and colleagues [53] found that the pro-longevity effects of rapamycin and DR were additive. Global expression of a constitutively active S6K did not attenuate the IF-induced lifespan extension, whereas the lifespan-extending effects of rapamycin were abolished by this method [53]. Our results agree with findings in yeast and nematodes that IF-induced lifespan extension can act independently of TOR signaling [31, 32]. It should be noted that we would not recommend the 2:5 diet, cycles of 5 consecutive days of water-only fasting per week, in humans.

In summary, we have generated and optimized a reliable and robust regime of IF for lifespan extension in flies. We conclude that IF during early life can robustly increase lifespan in a TOR-independent manner and likely acts by preserving gut health. Importantly, the beneficial effects of short-term IF were long-lasting, indicating that even brief IF periods may have long-lasting health benefits, a phenomenon that should be subject to further investigation in mammals.

## STAR★Methods

### Key Resources Table

**Table:** 

REAGENT or RESOURCE	SOURCE	IDENTIFIER
**Antibodies**		

Mouse monoclonal anti-integrin βPS (myospheroid) antibody	Developmental Studies Hybridoma Bank	Cat# #*CF.*6G11 RRID:AB_528310
Rabbit polyclonal phospho-Histone H3 (Ser10) antibody	Cell Signaling Technology	Cat# 9701 RRID:AB_331535
Alexa Fluor 594 Donkey Anti-Rabbit IgG (H L) Antibody	Thermo Fisher Scientific	Cat# A21207 RRID:AB_141637
Alexa Fluor 488 Donkey Anti-Mouse IgG (H L) Antibody	Thermo Fisher Scientific	Cat# A21202 RRID:AB_141607

**Chemicals, Peptides, and Recombinant Proteins**		

Rapamycin	LC Laboratories	R-5000 CAS Number: 53123-88-9
FD&C Blue dye No.1	Fastcolors	CI: 42090 CAS Number: 3844-45-9
Paraquat (Methyl viologen dichloride hydrate)	Sigma-Aldrich	Cat# 856177
DDT (Dichlorodiphenyltrichloroethane)	Sigma-Aldrich	Cat# N11567
Lysozyme from chicken egg white	Sigma-Aldrich	Cat# L7651
Ribonuclease A from bovine pancreas	Sigma-Aldrich	Cat# R4875
Vectashield with DAPI	Vector Laboratories	Cat# H-1200

**Critical Commercial Assays**		

Triglyceride Infinity Reagent	Thermo Fisher Scientific	Cat# TR22421
Pierce BCA Protein Assay Kit	Thermo Fisher Scientific	Cat# 23227
QIAGEN DNeasy Blood & Tissue Kit	QIAGEN	Cat# 69504
Power SYBR Green PCR Master Mix	Thermo Fisher Scientific	Cat# 4368706

**Experimental Models: Organisms/Strains**		

*D. melanogaster*: *w*^*Dah*^	This lab	N/A
*D. melanogaster*: *w*^*1118*^	Bloomington Stock Center	3605 RRID:BDSC_3605
*D. melanogaster*: *UAS-S6K*^*STDETE*^	Bloomington Stock Center	6914 RRID:BDSC_6914
*D. melanogaster*: *Da-GAL4*	Bloomington Stock Center	55850 RRID:BDSC_55850
*D. melanogaster*: *ovo*^*D*^	Bloomington Stock Center	1309 RRID:BDSC_1309

**Oligonucleotides**		

Primer: Pan bacterial 16S F (341F) - 5′-CCTACGGGAGGCAGCAG-3′	[59]	N/A
Primer: Pan bacterial 16S R (534R) - 5′-ATTACCGCGGCTGCTGG-3′	[59]	N/A
Primer: *L. plantarum* 16S F - 5′-AGGTAACGGCTCACCATGGC-3′	[59]	N/A
Primer: *L. plantarum* 16S R - 5′- ATTCCCTACTGCTGCCTCCC-3′	[59]	N/A
Primer: *D. melanogaster* GAPDH F - 5′-TAAATTCGACTCGACTCACGGT-3′	[59]	N/A
Primer: *D. melanogaster* GAPDH R - 5′- CTCCACCACATACTCGGCTC-3′	[59]	N/A

**Software and Algorithms**		

ImageJ	ImageJ	https://imagej.nih.gov/ij/
Microsoft Excel	Microsoft	https://www.microsoft.com/en-gb/
GraphPad Prism	GraphPad	https://www.graphpad.com/scientific-software/prism/
Adobe Illustrator	Adobe	https://www.adobe.com/uk/products/illustrator.html
R statistics package	R Core Team	https://www.r-project.org/

**Other**		

Glycerol Standard Solution	Sigma-Aldrich	Cat# G7793
7 mL Bijou sample container	Sigma-Aldrich	Cat# Z645338
Microcaps glass capillaries 5 μL	CAMAG	Cat# 552-0043
Zeiss LSM 700 confocal laser scanning microscope	Zeiss	N/A
Plan-Apochromat 40x/1.3 Oil DIC M27	Zeiss	N/A
Leica M165C digital stereo microscope	Leica	N/A
Leica IC80 HD camera	Leica	N/A
Nanodrop 2000C spectrophotometer	Thermo Fisher Scientific	N/A
QuantStudio 6cFlex real-time PCR machine	Thermo Fisher Scientific	N/A

### Contact for Reagent and Resource Sharing

Further information and requests for resources and reagents should be directed and will be fulfilled by the Lead Contact, Linda Partridge (l.partridge@ucl.ac.uk).

### Experimental Model and Subject Details

The wild-type *Drosophila melanogaster* stock Dahomey was collected in 1970 in Dahomey (now Benin) and has since been maintained in large population cages with overlapping generations on a 12L:12D cycle at 25°C. The *white* Dahomey (*w*^*Dah*^) stock was derived by incorporation of the *w*^*1118*^ deletion into the outbred Dahomey background by successive backcrossing. Mutants and transgenes were backcrossed into *w*^*Dah*^ Wolbachia-positive strain for at least eight generations. Stocks were maintained and all experiments were conducted at 25°C on a 12h:12h light/dark cycle at constant 65% humidity using standard sugar/yeast/agar (SYA) medium unless otherwise stated [39].

### Method Details

#### Lifespan assay

Flies were reared at a standard density before being used for lifespan experiments. Crosses were set up in cages with grape juice agar and a small amount of live yeast paste for < 22-hr, then embryos were collected in PBS and squirted into bottles at ∼18 μl per bottle to achieve standard density (18 μl embryos = ∼300 flies per bottle) [39, 60]. All experiments were performed with flies (females and males) that were allowed 48 h to mate after emerging as adults. Flies were subsequently lightly anaesthetized with CO_2_, sorted into single sexes and counted at 10 or 15 per vial, with 10-20 vials per condition. The minimum number of flies set up per condition was 150. Agarose was used during the fasting periods as a water source. Rapamycin (R-5000, LC Labs, Woburn, MA, US) was dissolved in ethanol and added to food to make a final concentration of 200 μm. For control food ethanol alone was added. In all cases, flies were transferred to fresh food at least three times a week, at which point deaths/censors were scored. Censored flies were excluded from the final analysis. Where possible, lifespans were repeated at least twice. Details of statistical evaluations, and the number of flies per condition are provided in the figure legends. Log-rank tests of survivorship curves were performed in Excel (Microsoft), and Cox proportional hazards analysis for multiple comparisons was performed in R statistics package (R Core Team).

#### Fecundity assay

Eggs were collected over 24-hour periods at several time-points during lifespan experiments. Fecundity was not measured blind due to the compositional difference in the media the flies were placed on (i.e., opaque food media versus agarose media). The number of eggs laid per vial at each time point was counted. 20 vials were counted per condition. No vials were excluded from the analysis. Each vial contained 10-15 flies. Data are reported as the mean number of eggs laid per female fly per 24h ± SEM.

#### Lipid assay

For triacylglyceride (TAG) content quantification, two whole female flies were homogenized in 0.05% Tween20 according to [61]. 10 replicates were used per condition. TAG content was quantified using the Triglyceride Infinity Reagent (ThermoScientific) using Glycerol standards (Sigma). Protein content was determined using the BCA protein assay reagent (Pierce). Student’s t test (Excel) was used to assess statistical difference between two conditions, while one-way ANOVA (GraphPad) was used for > 2 conditions.

#### Capillary Feeder (CAFE) assay

A 7 mL bijou vial filled with 1 mL of (1%) agar, to ensure humid conditions, was sealed with Parafilm (Alpha Laboratories Ltd, Hampshire, UK) after a single fly was added. Four holes in the Parafilm that were equally spaced apart, were made using a 26-gauge needle to ensure adequate air circulation. Through the Parafilm was inserted a truncated 200 μl pipette tip which held a graduated 5 μl disposable glass capillary tube (Camag, Muttenz, Switzerland) containing liquid food (5% sucrose (w/v), 2% BD Bacto yeast extract (w/v)) supplemented with 0.5 mg/mL FD&C blue dye no. 1 (Fastcolors) to aid measurement of feeding. For all experiments, a mineral oil overlay (0.1 μl) was used to minimize evaporation. Food ingestion was measured every 24-hr. Each experiment included an identical, CAFE chamber without flies to determine evaporative losses (typically 10% of ingested volumes), which were subtracted from experimental readings [62]. A minimum of 24 individually housed flies per condition was used. CAFE assays were not performed blind. Flies that died during CAFE experiments were censored and excluded from the final analysis. Student’s t test (Excel) was used to assess statistical difference between two conditions at a specified time point, while two-way ANOVA (GraphPad) was used for 2 conditions over time.

#### Stress assays

Flies were reared and housed as for lifespan experiments. 6-week old flies were transferred to agar (for starvation assay), or food supplemented with either 20 mM Paraquat (Methylviologen, from Sigma) for oxidative stress assay, or 0.06% (w/v) DDT (Dichlorodiphenyltrichloroethane, from Sigma) for xenobiotic stress assay. Stress assays were scored at least 3 times per day throughout the assay. Censored flies were excluded from the final analysis. Where possible, stress survival assays were repeated at least twice. Details of statistical evaluations, and the number of flies per condition are provided in the figure legends. Log-rank tests of survivorship curves were performed in Excel (Microsoft).

#### Immunohistochemistry and imaging of the gut

Guts were dissected from live flies in ice cold PBS and immediately fixed in 4% formaldehyde for 15 min, serially dehydrated in MeOH, stored at −20 °C, and subsequently stained. Guts were washed in 0.2% Triton-X / PBS, blocked in 5% bovine serum albumin / PBS, incubated in primary antibody overnight at 4 °C and in secondary for 2 hr at RT. Guts were mounted in mounting medium containing DAPI (Vectastain). 19 to 20 guts were analyzed per condition. Broken or damaged guts were excluded from the final analysis. Anti-βPS-integrin (1:10 of hybridoma supernatant (100 μg/ml, #*CF.*6G11), Developmental Studies Hybridoma Bank) was used to mark the outer muscles of the gut, and anti-phospho-Histone H3 (1:500, #9701) to stain mitotic cells (Cell Signaling, UK). Secondary antibodies: Alexa Fluor 594 donkey anti-rabbit ((A21207) Thermo Fisher Scientific, Waltham, MA) 1:1000; Alexa Fluor 488 donkey anti-mouse (A21202) 1:1000. The hybridoma developed by [63] was obtained from the Developmental Studies Hybridoma Bank developed under the auspices of the NICHD and maintained by The University of Iowa, Department of Biology, Iowa City, IA 52242. Images were captured with a Zeiss (UK) LSM 700 confocal laser scanning microscope using a 10x or 40x oil-immersion objective. Images were taken using the 10x or 40x objective as stacks and are shown as maximum intensity projections of the complete stack. The same size stacks were taken for experimental and control samples. All images for one experiment were taken at the same settings.

#### Quantifying ISC activity

ISCs from whole guts were visually counted on the microscope. Counts were then averaged and plotted in GraphPad. 19-20 guts were counted per condition. One-way ANOVA (GraphPad) was used to assess statistical difference between conditions.

#### Measuring gut length/width

Brightfield images of guts (with appropriate scale bars included) were taken on a Leica M165C microscope with a Leica IC80 HD camera, which were then analyzed using ImageJ. Using the Measure tool, guts were traced freehand and distances (lengths and widths) were recorded. 11-13 guts were measured for each condition. Measurements were not performed blind. Broken or damaged guts were excluded from the final analysis. One-way ANOVA (GraphPad) was used to assess statistical difference between conditions.

#### Scoring gut pathology

Cross-section gut images were randomized and scored blind. Pathologies were binned into scaled categories and quantified, n = 12/13 per condition. R2 and R4 categories were defined as follows: I = non-pathological, single layer epithelium. II = sporadic pathology of small nuclei ‘nests’ without significant disruption to the epithelium; III = widespread pathology, majority of epithelium has several layers of nuclei; IV = widespread pathology plus clear tumor formation.

#### Gut barrier function (Smurf) assay

Gut barrier efficiency at day 70 was analyzed by placing flies on blue food (minimum 150 flies per condition) prepared using 2.5% (w/v) FD&C blue dye no. 1 (Fastcolors) for 24 hr before the Smurf phenotype was scored. After 24-hr, flies were scored as ‘smurf/non-smurf’ by the presence or absence of blue dye outside of the gut (i.e., visible in the haemolymph). A minimum of 240 flies were assessed per condition. The breakdown of the smurf numbers is provided in the figure. Fisher’s exact (GraphPad) was used to assess statistical difference between conditions.

#### 16S qPCR quantification of bacterial load

For each biological replicate, we extracted DNA from samples using the QIAGEN DNeasy Blood and Tissue kit using the following modified protocol [64]. Flies were sterilized with 70% ethanol to remove exterior bacteria then 180 μL lysis buffer (20 mM Tris-HCl pH 8.0, 2 mM EDTA pH 8.0, 1.2% Triton X-100, and 20 mg/ml fresh lysozyme from chicken egg (Sigma, L7651)), and 200 μL QIAGEN Buffer AL was added. Following lysis using a Kontes pellet pestle, we added 20 μL proteinase K (QIAGEN) and incubated the samples at 56°C for 3 hr. To ensure there would be no remaining RNA in our sample, we added 10 μg/ml RNase A (Sigma, R4875) and incubated at 37°C for 30 min. We then added 200 μL EtOH and proceeded with the standard QIAGEN spin-column protocol.

We performed quantitative real-time PCR on total genomic DNA to determine the ratio of bacterial to fly DNA in each sample. We used two flies per biological replicate, and eight biological replicates per condition. qPCR was carried out by using Power SYBR Green PCR Master Mix (Thermo Fisher Scientific) on QuantStudio 6 Flex real-time PCR (Applied Biosystems, Thermo Fisher Scientific, Waltham, MA). The following primer sequences (Eurofins, UK) were used in the analysis [59]: Pan bacterial 16S (341F + 534R): 5′-CCTACGGGAGGCAGCAG-3′, 5′-ATTACCGCGGCTGCTGG-3′; *L. plantarum* 16S: 5′-AGGTAACGGCTCACCATGGC-3′, 5′- ATTCCCTACTGCTGCCTCCC-3′; *D. melanogaster* GAPDH: 5′-TAAATTCGACTCGACTCACGGT-3′, 5′- CTCCACCACATACTCGGCTC-3′. Data were plotted in GraphPad and Student’s t test was used to assess statistical difference between conditions.

### Quantification and Statistical Analysis

Microsoft Excel and GraphPad Prism were used for graphic representation and statistical analysis. Data were grouped for each genotype and the mean (+/− SEM) calculated. Log-rank, Cox proportional hazard, Fisher’s exact, Student’s t test, analysis of variances (ANOVA) and Tukey’s HSD (honestly significant difference) post hoc analyses were performed. Statistical analyses were performed in Excel (Microsoft) or Prism (GraphPad, La Jolla, CA), except for Cox Proportional Hazards which were performed in R (R Core Team). A statistical difference of p < 0.05 was regarded as significant. Statistical tests and significance levels are indicated in each figure legend.

## Supplemental Information

Document S1. Figures S1–S4

Document S2. Article plus Supplemental Information
