# Supplementary material for: Short-Term, Intermittent Fasting Induces Long-Lasting Gut Health and TOR-Independent Lifespan Extension
Source: Curr Biol. 2018 Jun 4;28(11):1714–1724.e4. doi: 10.1016/j.cub.2018.04.015 (PMC5988561; doi:10.1016/j.cub.2018.04.015)
Supplement: Document S1. Figures S1–S4 [file mmc1.pdf]

Current Biology, Volume *28*

Supplemental Information

Short-Term, Intermittent Fasting Induces Long-Lasting Gut Health and TOR-Independent Lifespan Extension

James H. Catterson, Mobina Khericha, Miranda C. Dyson, Alec J. Vincent, Rebecca Callard, Steven M. Haveron, Arjunan Rajasingam, Mumtaz Ahmad, and Linda Partridge

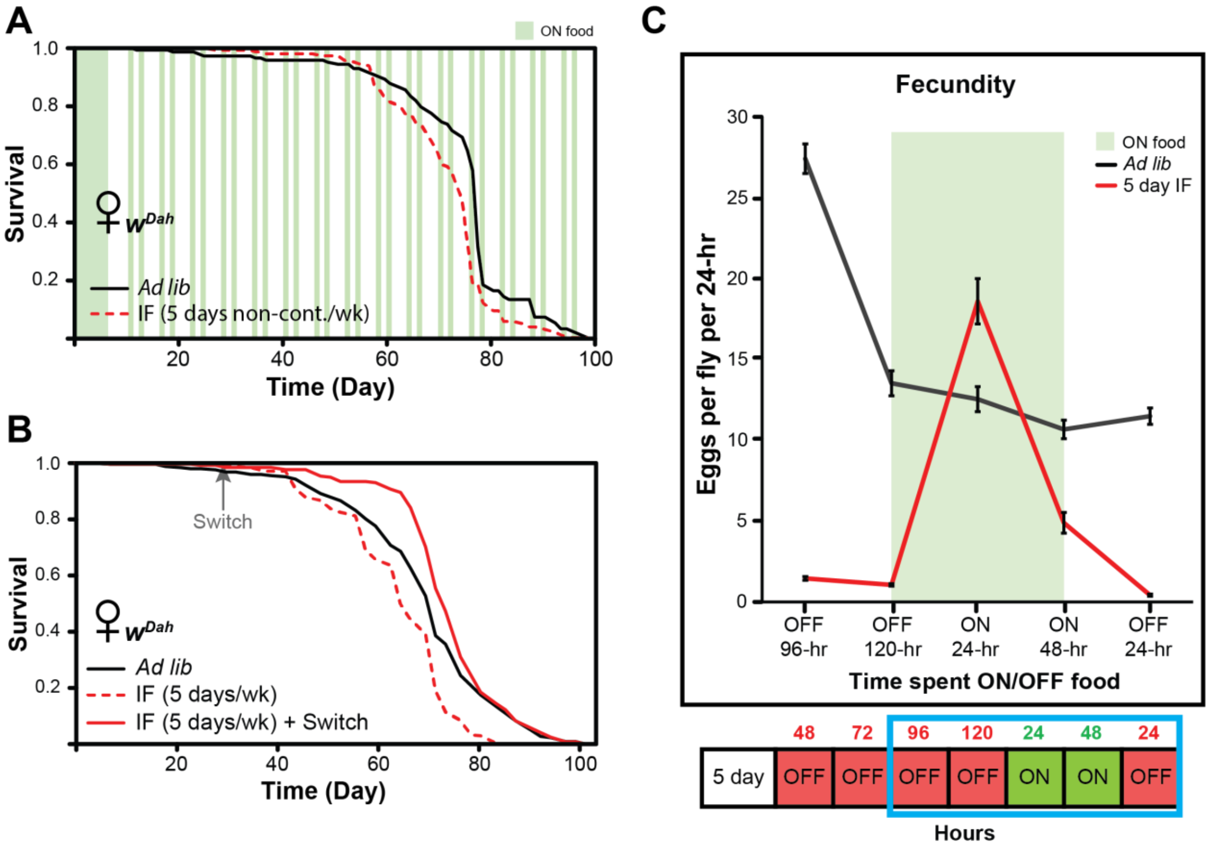

A
ON food
1.0
0.8
0.6
0.4
0.2
Survival
♀ w^Dah
Ad lib
IF (5 days non-cont./wk)
20
40
60
80
100
Time (Day)
B
1.0
0.8
0.6
0.4
0.2
Survival
Switch
♀ w^Dah
Ad lib
IF (5 days/wk)
IF (5 days/wk) + Switch
20
40
60
80
100
Time (Day)
C
Fecundity
ON food
Ad lib
5 day IF
Eggs per fly per 24-hr
30
25
20
15
10
5
0
OFF 96-hr
OFF 120-hr
ON 24-hr
ON 48-hr
OFF 24-hr
Time spent ON/OFF food
48
72
96
120
24
48
24
5 day
OFF
OFF
OFF
OFF
ON
ON
OFF
Hours

Figure S1. IF prolongs lifespan, but only after a Switch to *ad lib* food. Related to Figure 1.

(**A**) Fasting for 5 non-consecutive days per week significantly ($p = 4.5 \times 10^{-06}$, log-rank test) shortened lifespan compared to *ad lib* controls. n>160 flies per condition. (**B**) Switching flies fasted for 5 consecutive days per week after 30 days of the regime significantly increased lifespan compared to *ad lib* controls ($p = 0.00995$, log-rank test). n>245 flies per condition. (**C**) The schematic shows the days on which eggs were counted. Eggs were counted from day 8 to day 12 of adult life. Egg-laying was abolished in fasted flies, but then increased to above *ad lib* levels after 24-hr on food. Note that egg-laying dropped back below *ad lib* levels after 48-hr on food. Eggs were counted every 24-hr from 20 vials of 10 flies (n = 200) per condition. Data are shown as mean ± SEM.

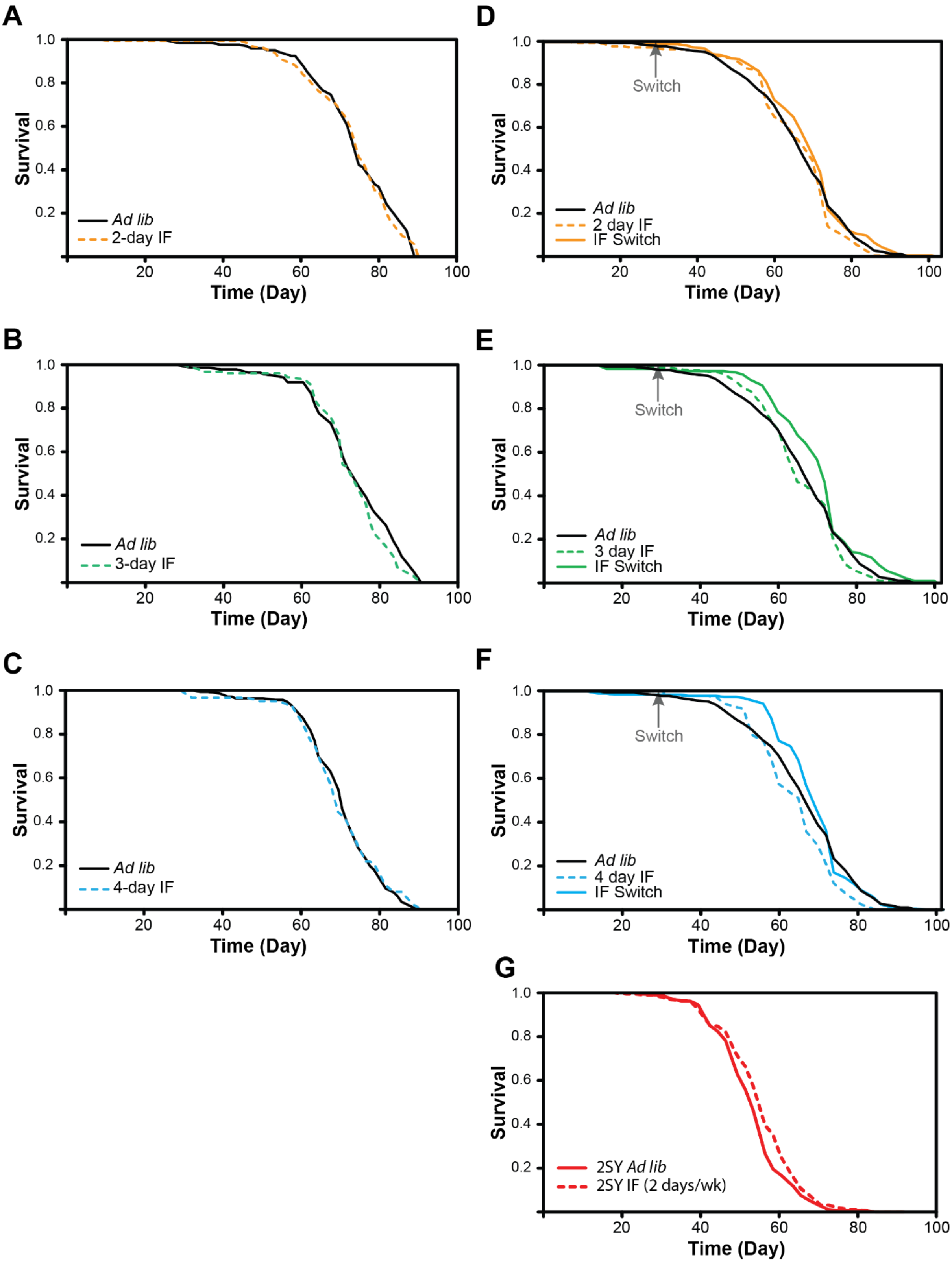
A
B
C
D
E
F
G
Survival
Time (Day)
Ad lib
2-day IF
3-day IF
4-day IF
2 day IF
3 day IF
4 day IF
IF Switch
Switch
2SY Ad lib
2SY IF (2 days/wk)

Figure S2. IF for 2/3/4 days per week is not enough to robustly extend lifespan. Related to Figure 1.

(**A**) Fasting for 2 consecutive days per week did not affect lifespan of flies when compared to their *ad lib* controls. n>140 flies per condition. (**B**) 3-day IF throughout life did not affect lifespan when compared to their *ad lib* controls. n>135 flies per condition. (**C**) 4-day IF throughout life did not affect lifespan when compared to their *ad lib* controls. n>135 flies per condition. (**D**) Switching flies fasted for 2 consecutive days per week after 30 days of the regime did not affect lifespan compared to *ad lib* controls. n>195 flies per condition. (**E**) Switching flies fasted for 3 consecutive days per week after 30 days of the regime significantly increased lifespan compared to *ad lib* controls ($p = 0.0057$, log-rank test). n>195 flies per condition. (**F**) Switching flies fasted for 4 consecutive days per week after 30 days of the regime did not affect lifespan compared to *ad lib* controls. n>195 flies per condition. (**G**) When flies were fed 2SY food, fasting for 2 days per week significantly ($p = 0.020$, log-rank test) increased lifespan compared to *ad lib* controls. n>194 flies per condition.

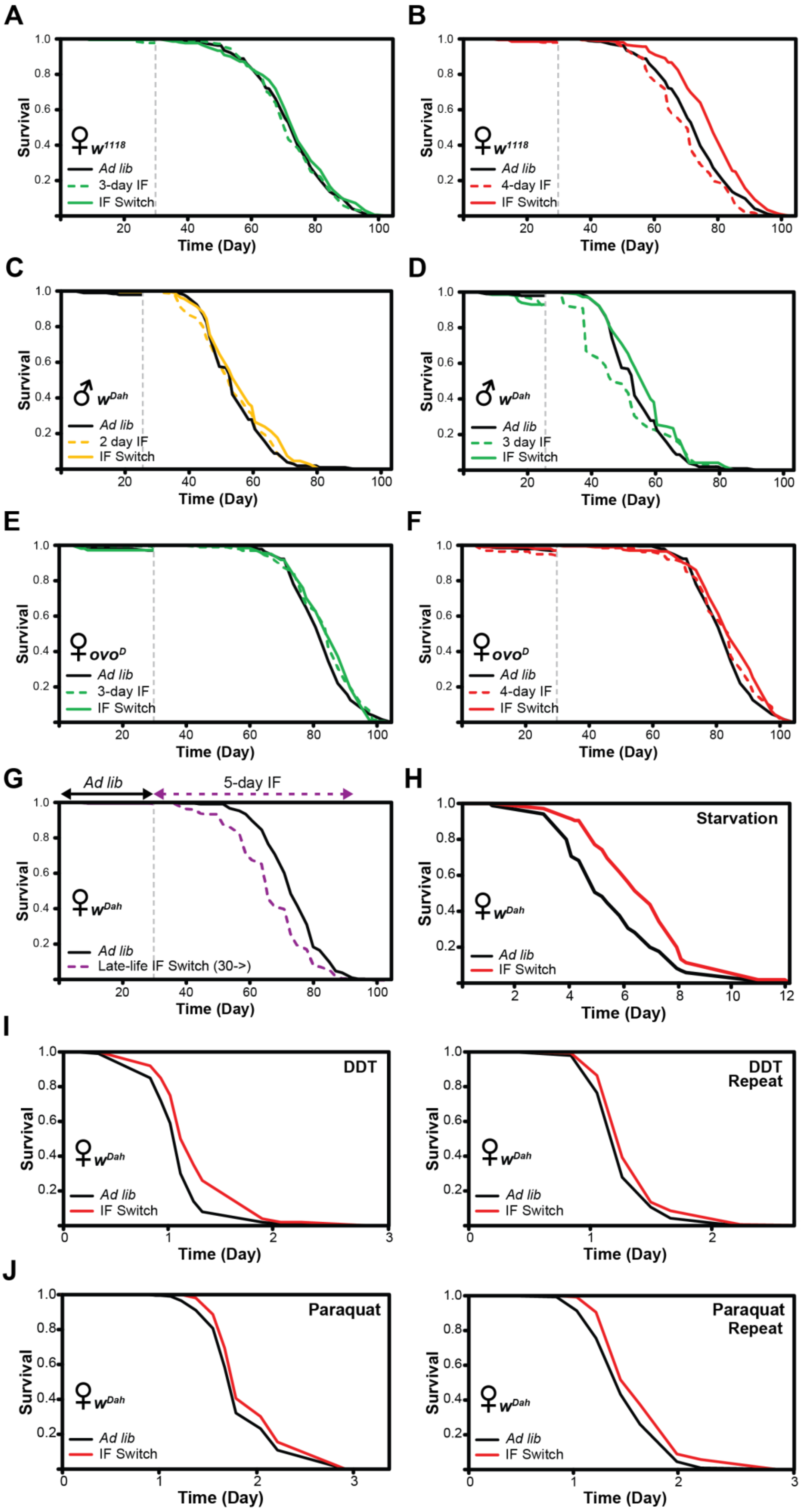

A
Survival
Time (Day)
♀ w1118
Ad lib
3-day IF
IF Switch
B
♀ w1118
Ad lib
4-day IF
IF Switch
C
♂ wDah
Ad lib
2 day IF
IF Switch
D
♂ wDah
Ad lib
3 day IF
IF Switch
E
♀ ovoD
Ad lib
3-day IF
IF Switch
F
♀ ovoD
Ad lib
4-day IF
IF Switch
G
Ad lib
5-day IF
♀ wDah
Ad lib
Late-life IF Switch (30->)
H
Starvation
♀ wDah
Ad lib
IF Switch
I
DDT
♀ wDah
Ad lib
IF Switch
DDT Repeat
J
Paraquat
♀ wDah
Ad lib
IF Switch
Paraquat Repeat

Figure S3. IF leads to enhanced stress resistance. Related to Figure 1 and 2.

(**A**) Switching w^{1118} flies fasted for 3 consecutive days per week after 30 days of the regime did not affect lifespan compared to *ad lib* controls. n>210 flies per condition. (**B**) Switching w^{1118} flies fasted for 4 consecutive days per week after 30 days of the regime significantly extended lifespan compared to *ad lib* controls ($p = 5.9 \times 10^{-06}$, log-rank test). n>205 flies per condition. (**C**) Switching male flies fasted for 2 consecutive days per week after 25 days of the regime did not affect lifespan compared to *ad lib* controls (p = 0.13, log-rank test). n>180 flies per condition. (**D**) Switching male flies fasted for 3 consecutive days per week after 25 days of the regime increased lifespan compared to *ad lib* controls (p = 0.020, log-rank test). n>180 flies per condition. (**E**) Switching ovo^{D} flies fasted for 3 consecutive days per week after 30 days of the regime did not affect lifespan compared to *ad lib* controls. n>195 flies per condition. (**F**) Switching ovo^{D} flies fasted for 4 consecutive days per week after 30 days of the regime significantly extended lifespan compared to *ad lib* controls (p = 0.013, log-rank test). n>190 flies per condition. For display purposes, the *ad lib* condition is the same data in each separate graph (solid black line). (**G**) Switching w^{Dah} flies to 5-day IF after 30 days of *ad lib* food significantly shortened lifespan compared to *ad lib* controls ($p = 2.3 \times 10^{-07}$, log-rank test). n>220 flies per condition. Grey dashed line indicates the 'switch' point, after which the lifespan curves are 'reset' and deaths before this point become censored. (**H**) 42-day old post-IF females exhibited significantly enhanced resistance to starvation compared to *ad lib* controls (p = 0.00031, log-rank test). n = 90-100 flies per condition. (**I**) Two independent replicate experiments demonstrated that 42-day old IF Switch flies exhibited significantly enhanced resistance to DDT stress compared to *ad lib* controls (p = 0.00027, and p = 0.016 respectively, log-rank test). n = 100 or 150 flies per condition, respectively. (**J**) Two independent replicate experiments demonstrated that 42-day old IF Switch flies exhibited a small but significant resistance to paraquat stress compared to age-matched *ad lib* controls (p = 0.018, and p = 0.0031 respectively, log-rank test). n = 225 or 150 flies per condition, respectively.

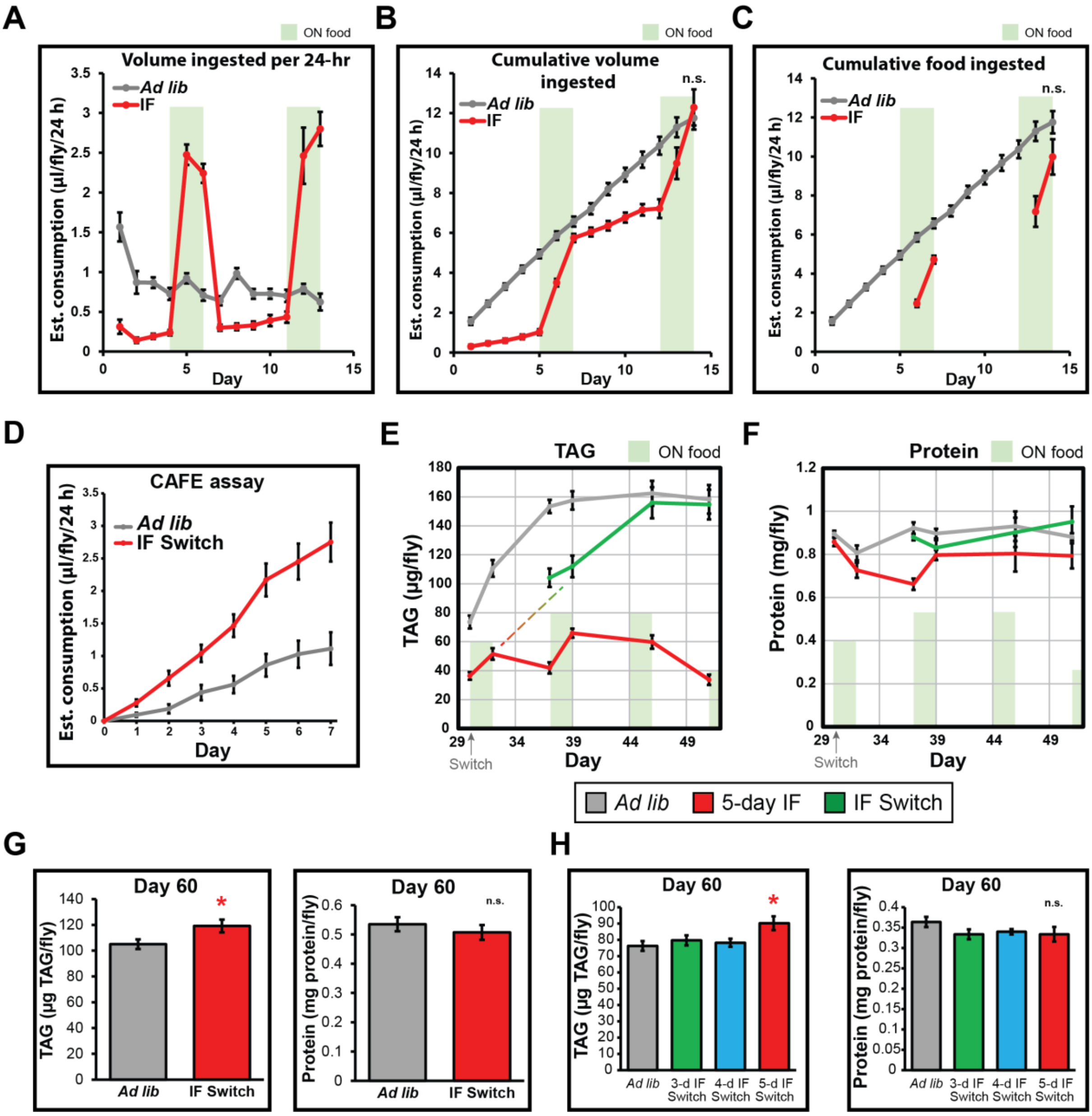

A
ON food
Volume ingested per 24-hr
Ad lib
IF
Est. consumption (µl/fly/24 h)
Day
B
ON food
Cumulative volume ingested
n.s.
Ad lib
IF
Est. consumption (µl/fly/24 h)
Day
C
ON food
Cumulative food ingested
n.s.
Ad lib
IF
Est. consumption (µl/fly/24 h)
Day
D
CAFE assay
Ad lib
IF Switch
Est. consumption (µl/fly/24 h)
Day
E
TAG
ON food
TAG (µg/fly)
Switch
Day
F
Protein
ON food
Protein (mg/fly)
Switch
Day
Ad lib
5-day IF
IF Switch
G
Day 60
*
TAG (µg TAG/fly)
Ad lib
IF Switch
Day 60
n.s.
Protein (mg protein/fly)
Ad lib
IF Switch
H
Day 60
*
TAG (µg TAG/fly)
Ad lib
3-d IF Switch
4-d IF Switch
5-d IF Switch
Day 60
n.s.
Protein (mg protein/fly)
Ad lib
3-d IF Switch
4-d IF Switch
5-d IF Switch

Figure S4. Examining the effects of IF on feeding behaviour, and triglyceride levels in females. Related to Figure 1.

(**A-C**) Measurement of feeding behavior over 14 days using the capillary feeder (CAFE) assay during IF. (**A-B**) During the 2-day 'fed' period, ingestion was dramatically increased compared to *ad lib* controls. (**C**) At the end of the 14-day CAFE assay, cumulative food ingestion was not significantly different compared to *ad lib* controls ($p = 0.081$, Student's t-test). It should be noted, however, that mortality was high in the IF condition, with 8 flies alive at the last time point (compared to 17 in the *ad lib* condition). For the CAFE assay, food consumption was measured for 24 flies per condition every 24-hr. During the fasting period, fasted flies were given the same blue dye but with water instead of yeast/sugar. Data are shown as mean ± SEM. (**D**) Measurement of feeding behavior using the CAFE assay. From day 37 onwards (i.e. after a full week back on food), IF Switch flies consumed significantly more food over the week compared to *ad lib* controls ($p < 0.0001$, two-way ANOVA). Food consumption was measured for 24 flies per condition every 24-hr. Data are shown as mean ± SEM. (**E-F**) Measurement of triacylglyceride (TAG) and protein levels from day 30. (**E**) TAG levels were significantly reduced ($p < 0.0001$, two-way ANOVA) in 5-day IF flies compared to *ad lib* controls from the first measurement at day 30, while TAG levels in *ad lib* flies increased over time. TAG levels in IF Switch flies did not fully recover to *ad lib* levels after 1 week on *ad lib* food. TAG levels in IF Switch flies recovered to *ad lib* levels from day 46 (i.e. 2-weeks after the switch to *ad lib* food). Dashed coloured line indicates the TAG increase in IF Switch flies from day 30. (**F**) Protein levels were not significantly different in 5-day IF flies compared to *ad lib* controls ($p > 0.05$, two-way ANOVA). Protein levels in IF Switch flies were also not significantly different compared to *ad lib* controls ($p > 0.05$, two-way ANOVA). TAG and protein levels were from whole female flies. For each data point condition, ten biological replicates (2 flies per replicate) were measured. Data are shown as mean ± SEM. (**G**) Measurement of TAG and protein levels from 60-day old females (i.e. 30-days post-IF). TAG levels were significantly higher in IF Switch flies compared to *ad lib* controls ($p = 0.032$, Student's t-test), while protein levels were not different between conditions. For each data point condition, ten biological replicates (2 flies per replicate) were measured. Data are shown as mean ± SEM. (**H**) TAG and protein levels at day 60 from 3-day, 4-day, and 5-day IF Switch flies. TAG levels were significantly higher in 5-day IF Switch flies alone compared to the other conditions ($p = 0.025$, one-way ANOVA), while protein levels were not different between conditions. For each data point, ten biological replicates (2 flies per replicate) were measured. Data are shown as mean ± SEM.
